# Anthrax Vaccination, Gulf War Illness, and Human Leukocyte Antigen (HLA)

**DOI:** 10.3390/vaccines12060613

**Published:** 2024-06-04

**Authors:** Lisa M. James, Adam F. Carpenter, Brian E. Engdahl, Rachel A. Johnson, Scott M. Lewis, Apostolos P. Georgopoulos

**Affiliations:** 1 The GWI and HLA Research Groups, Brain Sciences Center, Department of Veterans Affairs Health Care System, Minneapolis, MN 55417, USAomega@umn.edu (A.P.G.); 2 Department of Neuroscience, University of Minnesota Medical School, Minneapolis, MN 55455, USA; 3 Department of Psychiatry, University of Minnesota Medical School, Minneapolis, MN 55455, USA; 4 Department of Neurology, University of Minnesota Medical School, Minneapolis, MN 55455, USA; 5 Department of Psychology, University of Minnesota Medical School, Minneapolis, MN 55455, USA

**Keywords:** anthrax vaccination, Gulf War illness (GWI), chronic multi-symptom illness (CMI), human leukocyte antigen (HLA), binding affinity

## Abstract

We report on a highly significant, positive association between anthrax vaccination and occurrence of Gulf War Illness (GWI) in 111 Gulf War veterans (42 with GWI and 69 controls). GWI was diagnosed in 47.1% of vaccinated veterans but only in 17.2% of non-vaccinated veterans (Pearson *χ*^2^ = 7.08, *p* = 0.008; odds ratio = 3.947; relative risk = 2.617), with 1.6x higher GWI symptom severity in vaccinated veterans (*p* = 0.007, F-test in analysis of covariance). Next, we tested the hypothesis that the susceptibility to GWI following anthrax vaccination could be due to inability to make antibodies against the anthrax protective antigen (PA), the key protein contained in the vaccine. Since the first step in initiating antibody production would be the binding of PA peptide fragments (typically 15-amino acid long [15-mer]) to peptide-binding motifs of human leukocyte antigen (HLA) Class II molecules, we assessed the binding-motif affinities of such HLA specific molecules to all linear 15-mer peptide fragments of the anthrax PA. We identified a total of 58 HLA Class II alleles carried by the veterans in our sample and found that, of those, 18 (31%) were present in the vaccinated group that did not develop GWI but were absent from the vaccinated group who developed GWI. Remarkably, in silico analyses revealed very high binding affinities of peptide-binding motifs of those 18 HLA alleles with fragments of anthrax vaccine PA, leading to the successful production of anti-PA antibodies. Conversely, the absence of these protective HLA alleles points to a reduced ability to develop antibodies against PA, thus resulting in harmful PA persistence and development of GWI.

## 1. Introduction

### 1.1. Anthrax Vaccine 

Vaccines against the deadly disease “anthrax” caused by *Bacillus anthracis* (*B. anthracis*) have been developed to protect from potential exposure to the pathogen [1]. The formulations of licensed anthrax vaccines vary across countries [2], although all such vaccines contain the antigenic material of *B. anthracis.* Anthrax Vaccine Adsorbed (AVA; BioThrax^®^) [3], which is licensed in the United States (US) and was administered to US Gulf War (GW) veterans, contains proteins including the 83 kDa protective antigen (PA) of *B. anthracis* and an aluminum adjuvant to enhance immunogenicity. The original formulation developed in 1963 contained strain V770-NPl-R [4], but the strain of *B. anthracis* used in the current AVA formulation is unspecified. Additional details about the preparation of AVA can be found elsewhere [3]. It is worth noting that the composition of AVA has not been fully characterized, in contrast to the composition of a very similar anthrax vaccine licensed in the United Kingdom (UK), which has been characterized precisely using liquid chromatography–tandem mass spectrometry and found to contain “at least 138 *B. anthracis* proteins, including PA (65%), Lethal Factor (8%) and Edema Factor (3%)” [5]. Given that these proteins may have adverse effects, the detailed characterization of the AVA composition is urgently needed. As discussed in a recent review [1], the production of new generation anthrax vaccines using recombinant *B. anthracis* antigens would yield a “clean” vaccine without impurities stemming out of the current production process. The anthrax vaccine has more short-term side effects than other administered vaccines and unknown long-term side effects [1]. 

### 1.2. Gulf War Illness (GWI)—Chronic Multi-Symptom Illness (CMI)

Gulf War illness (GWI) is a systemic and debilitating condition of unknown etiology affecting one-third of the 1990-91 Gulf War (GW) era U.S. veterans. Common symptoms include fatigue, joint and muscle pain, and neurological/cognitive/mood dysfunction, in addition to gastrointestinal, respiratory, and skin symptoms [6,7,8]. GWI is now considered a chronic multi-symptom illness (CMI) [8,9,10,11,12,13]. Various factors have been implicated in GWI etiology, including toxic exposures [14] and immunogenetic deficits [15].

### 1.3. Human Leukocyte Antigen (HLA)

The rationale of vaccination is to induce the production of antibodies against the antigen contained in the vaccine, so as to neutralize that antigen in the event of a future exposure. The human immune response to vaccines and other foreign antigens is basically governed by the individual’s HLA composition. Specifically, each individual possesses 12 classical HLA genes, including six Class I (HLA-A, B, C) and six Class II (HLA-DR, DQ, DP) genes that code for cell surface molecules which are instrumental in immune surveillance and the elimination of foreign antigens [16]. HLA Class I molecules form a complex with small peptides (typically of nine amino acids) from proteolytically degraded cytosolic antigens that is exported to the cell surface for presentation to CD8 + cytotoxic T lymphocytes to signal cell destruction. HLA Class II molecules bind with, and export, larger peptides (typically of 15 amino acids) derived from endocytosed exogenous antigens to CD4 + T lymphocytes, facilitating antibody production by B cells and the ensuing humoral adaptive immunity. In both cases, successful antigen elimination requires that epitopes derived from foreign antigens are capable of binding to HLA molecule-motifs with sufficient affinity to promote an immune response. HLA is the most highly polymorphic region of the human genome [17], and even single amino acid differences can alter the binding groove and, consequently, binding affinity [18]. In the absence of successful HLA–epitope binding, the antigen may persist, thereby causing a host of deleterious downstream effects including inflammation and tissue damage [19]. 

### 1.4. Anthrax Vaccination and HLA

Given that each individual carries only six HLA Class II alleles involved in initiating antibody production, it is not surprising that there is substantial variation in the human immune response to vaccines in general [20] and to the anthrax vaccine in particular [1,5]. With respect to the latter, observations in human vaccinees [5] have been complemented by the results of in silico studies [21] where HLA Class II alleles previously identified as protective against GWI [15] were also found to possess a very high binding affinity with the anthrax vaccine PA [21]. 

### 1.5. Anthrax Vaccination, GWI, and HLA

The anthrax vaccine, administered to Gulf War veterans to protect from harmful effects of possible anthrax-based biological warfare, has been controversial with regard to its role in GWI [1]. Recent studies have furnished evidence of the presence of PA in the serum of Gulf War veterans suffering from GWI [22,23] and demonstrated the apoptotic effects of this antigen on neural cell cultures [24], raising new concerns about the potential contribution of persistent PA to the systemic deleterious health impacts associated with GWI [19]. The persistence of PA in a subset of Gulf War veterans three decades after vaccination is presumed to be related to the absence of immunogenetic protection against PA [15]. That is, exposure to anthrax vaccination in those lacking HLA that can bind with sufficient affinity to PA may contribute to negative health effects due to the inability to make antibodies against the vaccine antigen and eliminate it. To that end, recent evidence from in vitro studies has documented that the detrimental effects of serum from veterans with GWI on neural cultures were substantially reduced when antibodies against PA were added to the culture [22].

### 1.6. The Present Study 

There were two major objectives of this study. First, to investigate the possible association of anthrax vaccination with GWI, and, second, to test the hypothesis that such a positive association would be partially due to lack of immunogenetic protection against the anthrax vaccine antigen.

## 2. Materials and Methods

### 2.1. Participants

Study participants included 111 (101 men, 10 women) GW veterans (age 57.6 ± 8.4 y, mean ± SEM) recruited between 2018 and 2021. GWI status was determined by self-reported symptoms and in consideration of relevant inclusionary and exclusionary criteria consistent with both the Centers for Disease Control [6] and Kansas [7] case definitions, as recommended by the Institute of Medicine [8]. The self-report evaluates symptoms in six domains including fatigue, pain, neurological/cognitive/mood, respiratory, gastrointestinal, and dermatologic. Only symptoms that began during or after the Gulf War, were rated as at least of moderate severity, and lasting greater than 6 months counted towards the symptom domain. The average across domains was calculated as a measure of overall symptom severity. A total of 42 veterans met the criteria for GWI and 69 were controls. Of the total participants, 82 veterans reported having received the anthrax vaccine. Study volunteers were compensated for their participation. This study’s protocol was approved by the Minneapolis Veterans Affairs Health Care System institutional review boards and informed consent was obtained prior to participating in this study in accordance with the Declaration of Helsinki. 

### 2.2. HLA Genotyping

DNA isolation was carried out from whole blood or saliva samples using commercially available kits (blood: ArchivePure cat. 2300730 from 5Prime distributed by Fisher Scientific or VWR; saliva: Oragene-Discover cat.OGR-500 coupled with prepIT purifier reagent cat. PT-L2P/ DNA Genotek Inc. Ottawa, ON, Canada). The purified DNA samples were sent to Histogenetics (http://www.histogenetics.com) for high-resolution HLA sequence-based typing (SBT; details are given in https://bioinformatics.bethematchclinical.org/HLA-Resources/HLA-Typing/High-Resolution-Typing-Procedures/ and https://bioinformatics.bethematchclinical.org/WorkArea/DownloadAsset.aspx?id=6482). Their sequencing DNA templates were produced by locus- and group-specific amplifications that included exon 2 and 3 for class I (A, B, C) and exon 2 for class II (DRB1, DQB1, and DPB1) and reported as antigen recognition site (ARS) alleles as per ASHI recommendation [25].

### 2.3. B. anthracis Protective Antigen (PA)

The amino acid (AA) sequence of PA, the active ingredient of the anthrax vaccine administered to GW veterans, was retrieved from the UniProt website (https://www.uniprot.org/uniprotkb/P13423/entry) on 27 April 2023. The PA consists of 764 amino acids whose sequence is given in Appendix A. The AA sequence was used to estimate HLA-PA binding affinity and immunogenicity in silico as follows.

### 2.4. PA-HLA Binding Affinities

#### 2.4.1. In Silico Estimation of Binding Affinity and Immunogenicity between PA and HLA Allele Motifs

**Figure 1:**
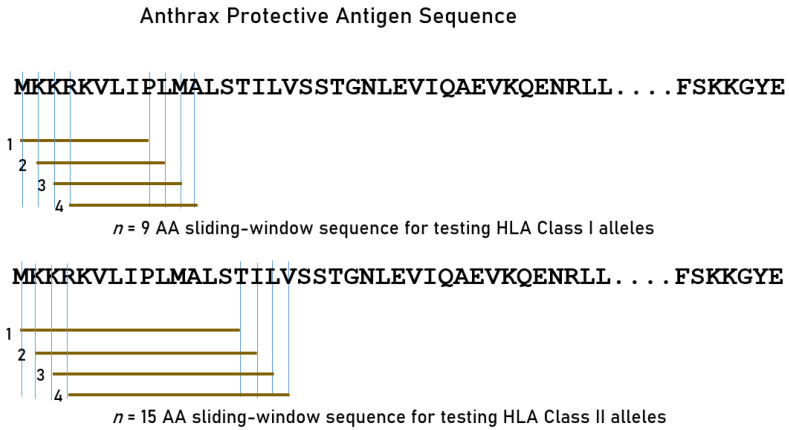
Schematic diagram to illustrate the exhaustive sliding window method for determining the estimated binding affinities of protective HLA alleles against PA 9-mer and 15-mer epitopes. AA, amino acid. See text for details.

We tested exhaustively the amino AA subsequences of PA using a sliding window approach (Figure 1) to partition the whole AA sequence of PA (Appendix A) into n-mers for PA-HLA analyses. Given the different groove lengths of HLA Class I and II molecules, we used a length of 9-mer for Class I and 15-mer for Class II alleles (Figure 1). For each n-mer, all possible subsequences were generated (number of subsequences = length of PA − n).

#### 2.4.2. Estimation of Binding Affinity and Immunogenicity of HLA Class I Alleles

We estimated the binding affinity and immunogenicity of all consecutive 9-mer AA subsequences of PA (N = 764 − 9 = 755) using the INeo-Epp method [26] [http://www.biostatistics.online/ineo-epp/neoantigen.php] on 6 September 2023. This method provides (a) a measure of binding affinity as a percentile rank (the smaller the better), and (b) a measure of immunogenicity as a continuous varying score. A combination of high binding affinity (<2 percentile rank) and a positive immunogenicity score >4 indicated a good, immunogenic binding to CD8+ (cytotoxic) lymphocytes.

#### 2.4.3. Estimation of Binding Affinity of HLA Class II Alleles

This analysis was limited to binding affinity only, because there is no method available (to our knowledge) that provides an immunogenetic score for HLA Class II alleles. The INeo-Epp method above works only for HLA Class I alleles; hence, we queried the IEDB database (www.iedb.org) to find the estimated binding affinity between all consecutive 15-mer PA epitopes (N = 764 − 15 = 749) and specific HLA Class II alleles. More specifically, binding affinity predictions were obtained on 16 August 2023 using the IEDB analysis resource NetMHCIIpan (ver. 4.1) tool [27] for HLA Class II predictions. A percentile rank <1 indicated good binding. The percentile rank was inverted, so that higher values indicated higher binding affinity.

### 2.5. Statistical Analyses

Standard statistical methods were used to analyze the data, including descriptive statistics, analysis of two-way tables, analysis of covariance (ANCOVA), etc. The IBM-SPSS package (version 29) was used for these analyses. All *p*-values reported are two-tailed.

## 3. Results

### 3.1. Anthrax Vaccination and GWI 

The analysis of the two-way table of event (absence or presence of GWI) x intervention (absence or presence of anthrax vaccination) (Table 1) revealed the following: (a) GWI was diagnosed 2.6x more frequently in veterans who had received the anthrax vaccine (45.1% vs. 17.2%, *p* = 0.008, Z = 2.661, Wald H0 test); (b) anthrax vaccination and GWI diagnosis were highly significantly associated (*p* = 0.008, Fisher’s Exact test; odds ratio = 3.947); (c) the relative risk of anthrax vaccination for GWI was 2.617; and (d) anthrax vaccination effected a 27.9% increase in absolute risk of GWI. These findings document a major and significant contribution of anthrax vaccination to GWI. Interestingly, 45/82 (55%) of anthrax-vaccinated veterans did not develop GWI. 

### 3.2. Anthrax Vaccination and GWI Symptom Severity

**Figure 2:**
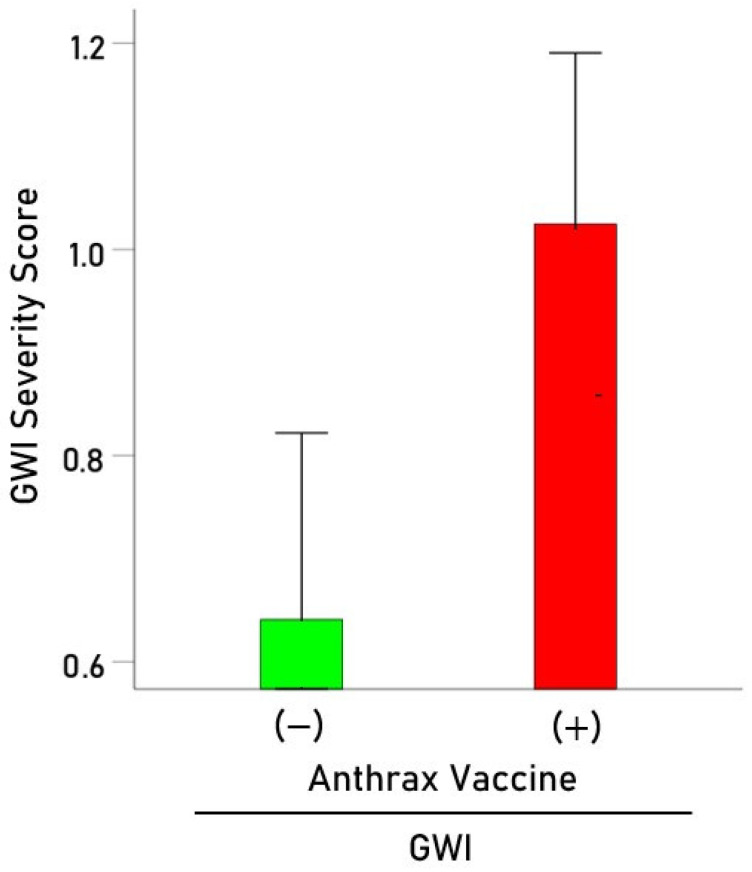
Average (± SEM) Kansas GWI severity score for GWI patients who did not receive (green) and those who received (red) anthrax vaccination. See text for details.

Among those veterans with GWI, we investigated the effect of anthrax vaccination on the severity of GWI symptoms by performing an analysis of covariance (ANCOVA), where the average Kansas score was the dependent variable, anthrax vaccination and deployment status were fixed factors, and age and sex were covariates. We found the following: (a) anthrax vaccination had a statistically highly significant effect on the overall GWI symptom severity (F[_1,37_] = 8.098, *p* = 0.007, ANCOVA); the average of scores in the six Kansas domains was 1.6x higher in the veterans who received the anthrax vaccine than in those who did not (Figure 2); (b) deployment status did not have a statistically significant effect on GWI symptom severity (F[_1,37_] = 0.974, *p* = 0.330); (c) there was no statistically significant effect of age (F[_1,37_] = 1.29, *p* = 0.263) or sex (F[_1,37_] = 0.859, *p* = 0.360). In summary, these findings document a positive association between anthrax vaccination and GWI and its detrimental effect on GWI symptom severity, independently of deployment status, age, and sex.

### 3.3. Role of HLA on Anthrax Vaccine–GWI Association

**Figure 3:**
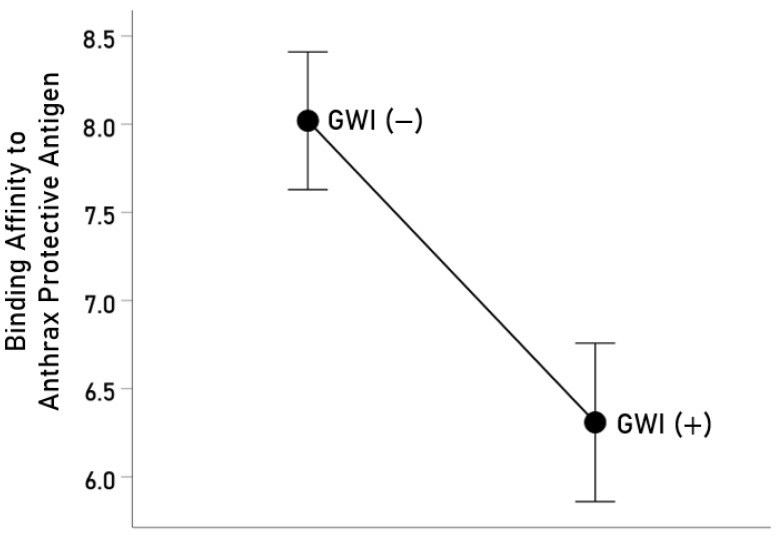
Mean (±SEM) estimated binding affinity of HLA Class II alleles in the GWI(−) and GWI(+) groups of anthrax-vaccinated veterans (see text for details).

It can be seen in Table 1 that of 82 veterans who reported receiving the anthrax vaccine, 37 developed GWI, whereas 45 did not. We hypothesized that the latter could be due to protection against GWI afforded by the genetic, adaptive immunity makeup of individual participants. Of those who were vaccinated, we were able to successfully genotype HLA in 35 GWI(+) and 45 GWI(−) veterans. In these 80 veterans, there were a total of 127 distinct HLA alleles (69 of Class I and 58 of Class II). Of the 69 Class I alleles, none yielded the requisite combination of percentile rank <2 and immunogenicity score > 4 for successful immunogenicity against PA, and hence they were not further analyzed. With respect to the Class II alleles, 18 occurred in the GWI(−) but were absent in the GWI(+) group; this proportion of 18/58 (31.0%) observed in the GWI(−) group was significantly different from 0% in the GWI(+) group (Z = 4.62, *p* < 0.00001, test of two proportions). These results suggest protection offered by these alleles against the offending anthrax vaccine PA. This hypothesis predicts that these alleles are associated with HLA cell-surface proteins that would bind with a high affinity to PA, the first necessary step for the successful formation of an *n*-mer and HLA molecule binding motif complex which would then engage the CD4+ lymphocytes for the subsequent production of anti-PA antibodies. We tested this prediction by estimating in silico the binding affinities of the 18 alleles that occurred only in the GWI(−) group and found that each allele indeed possessed a high binding affinity (percentile rank < 1) to PA (Table 2), thus lending strong support to the hypothesis. In addition, we compared the overall binding affinity of the 58 HLA Class II alleles between the GWI(−) [N = 45] and GWI(+) [N = 30] groups by analyzing the population of binding affinity estimates of <1 observed across the 58 alleles and the 749 15-nmer AA subsequences of PA. There were 2123 binding affinity values available for the GWI(−) and 1603 for the GWI(+) groups. We found a statistically significant overall higher binding affinity in the GWI(−) vs. GWI(+) group (Figure 3; *p* = 0.003, independent samples *t*-test, N = 3726). This result, in conjunction with the presence of 18 unique alleles in the GWI(−) group only, underscores the protective role of HLA genetic makeup against developing GWI in anthrax-vaccinated veterans and, conversely, points to a lack of sufficient HLA protection as a contributor to GWI following anthrax vaccine administration.

**Figure 4:**
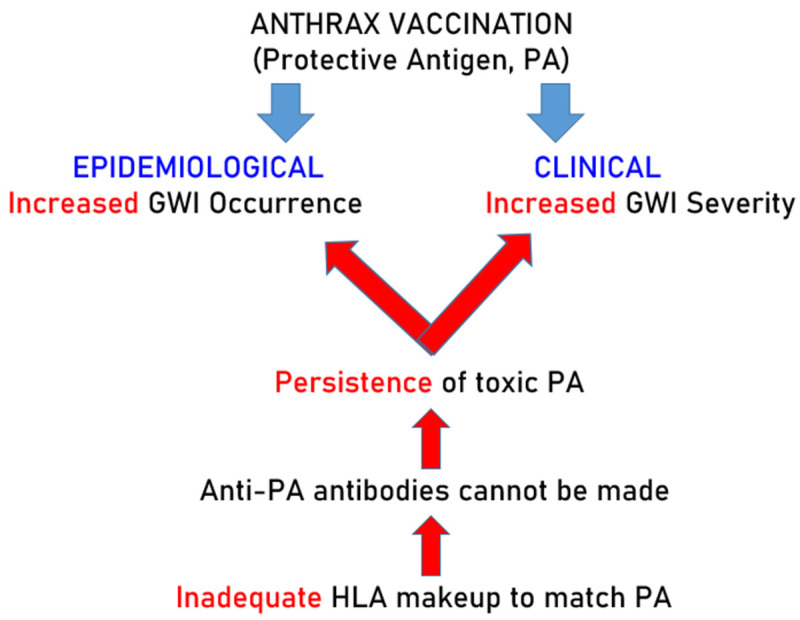
Schematic diagram to illustrate the experimental design and qualitative results. Red color indicates lack of protection against PA and its consequences.

The experimental design of this study and results are presented in Figure 4.

## 4. Discussion

The present findings documented a highly significant association between anthrax vaccination and GWI diagnosis, as well as significantly more severe symptoms in GW veterans who received the anthrax vaccine than those who did not. In addition, we identified a set of 18 HLA Class II alleles, involved in antibody production, which were present in the vaccinated, healthy veterans but were absent in the vaccinated veterans who developed GWI. These findings point to the persistence of the anthrax vaccine antigen as a major contributor to GWI and its severity, in keeping with the previous results on the PA-dependent apoptotic effect of GWI serum [22,23].

### 4.1. Anthrax Vaccination and GWI

The possible role of anthrax vaccination in GWI has long been debated [1,22,24,28,29,30,31,32]. Although the absence of vaccination information from Gulf War medical records has historically hampered efforts to conclusively link anthrax vaccination to the long-term health impacts affecting GW veterans [32], recent evidence pointing to the presence of anthrax vaccine PA in the serum of veterans with GWI [22,23] has renewed concerns about the potential harmful effects of anthrax vaccination [19].

Previous studies have documented the increased risk of GWI and poor health among those who reported receiving the anthrax vaccination [9,11,28]. It has been suggested that the significantly worse health status of veterans who reported having received the anthrax vaccination may be attributable to reporting biases [30]; however, substantial agreement between self-reported anthrax vaccination status and electronic recording of anthrax vaccination (where available) has been reported, indicating that service members accurately recall their anthrax vaccinations [31].

### 4.2. Persistence of Anthrax Vaccine PA Antigen and HLA

As mentioned above, the most likely explanation for the association between anthrax vaccination and GWI is the persistence of the anthrax vaccine antigen [19,22], which is toxic by itself [24]. The question then is, why the persistence? Since this antigen was administered, by definition, by the vaccine, and in multiple boosting doses, the simplest explanation is that it persists because it could not be removed. The main way of the PA being removed is by the production of antibodies against it [1,5,33]. Actually, it is the production of such antibodies that is the objective of vaccination, since those antibodies are expected to neutralize *B. anthracis* in case of exposure [34,35,36,37]. Now, the initial and necessary step for the production of antibodies is the successful formation of a complex between epitopes of the antigen and binding motifs of HLA Class II molecules [16]; this complex is transported to the cell membrane where it engages CD4+ lymphocytes, along the way to B cells where antibodies, specific to the antigen, are produced. It follows that if this antigen–HLA binding motif complex is not formed, no antibodies can be produced. Each individual carries six HLA Class II alleles, two of each of the three classical HLA genes (DPB1, DQB1, DRB1), and it is this set that determines whether antibodies to a specific antigen can be produced. In fact, variation in the allele composition of this set from individual to individual is a major determinant for a successful vaccination, namely the production of specific antibodies against the vaccine antigen. In the case of anthrax vaccination, the dependence of production of anti-PA antibodies on HLA has been documented [5,33,38]. Therefore, we analyzed the HLA composition of the vaccinated participants and identified 18 Class II HLA alleles which were present only in the group of veterans who did not develop GWI but were absent from the group that developed GWI. These findings suggest enhanced ability for antibody production against anthrax in vaccinated veterans who did not develop GWI. Now, as discussed above, effective antibody production hinges on high binding affinity between PA epitopes and HLA Class II allele binding motif complexes. Indeed, we estimated in silico this binding affinity and found that all 18 HLA Class II alleles that were exclusively present in the healthy vaccinated veterans were found to bind with very high affinity to PA. That finding, coupled with overall higher binding affinity of the 58 Class II HLA alleles in the healthy vaccinated veterans, suggests an enhanced ability to produce antibodies against PA in vaccinated veterans who did not develop GWI. In contrast, the absence of high binding affinity (i.e., protective) alleles may preclude successful elimination of PA, leading to GWI and the persistence of PA as documented in previous research [22,23].

Investigation of the specific mechanisms through which persistent PA (due to poor HLA-PA binding) contributes to GWI is beyond the scope of this paper. However, previous studies have documented the harmful effects of PA in vitro [24]. Specifically, the addition of PA to neuronal cell cultures resulted in decreased cell spreading and cell aggregation, increased apoptosis, compromised cell membrane permeability, disrupted cytoskeletal signaling pathways, and mitochondrial dysfunction. Notably, serum from veterans with GWI exhibited detrimental effects on cell cultures similar to those seen with PA that were substantially reduced by addition of specific antibodies against PA [22]. Finally, it is well established that persistent antigens may contribute to inflammation, autoimmunity, and apoptosis, each of which has been documented in GWI [39,40] in the absence of protective HLA [19].

### 4.3. Other Factors Contributing to GWI

The current findings highlight the potential contribution of anthrax vaccination to GWI in those lacking immunogenetic protection against PA. These findings, however, do not preclude other contributions to GWI. In fact, 17% of those who did not report having received the anthrax vaccine reported symptoms consistent with GWI. Thus, while insufficient HLA–PA binding may be a causative factor of GWI in some veterans, there are likely other contributory factors, potentially including any of the other vaccine antigens GW veterans received [23,32]. In addition to vaccines, several in-theater exposures such as burning pits, smoke from oil well fires, pesticides, organophosphates, sarin/cyclosarin nerve agents, pyridostigmine bromide, stress, and others have been associated with GWI [14,32]. Finally, it should be mentioned that stress has been shown to adversely affect the immune response to vaccinations in general [41], and bacterial vaccinations in particular [42].

## 5. Concluding Remarks and Current Status

**Figure 5:**
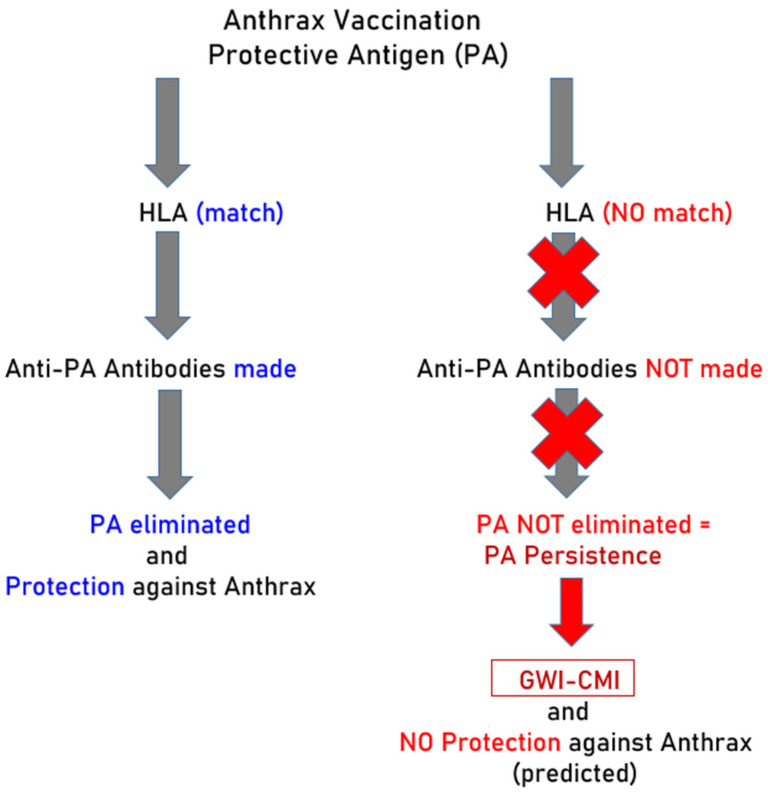
Schematic diagram to illustrate the interpretation of the results. Blue indicates effective antibody production and elimination of PA afforded by HLA-PA match. Red indicates lack of HLA protection against PA which prohibits antibody production and promotes PA persistence.

The cardinal characteristic of GWI is the involvement of multiple systems, hence its equivalent term “Chronic Multisystem Illness” (CMI) [8,9,10,11,12,13]. Remarkably, CMI has now been observed in veterans of subsequent Iraq/Afghanistan wars [43,44]. Originally, GWI was attributed to chemical exposures, including nerve gas, burning pits, insecticides, etc. [14,32]. Although these could be contributing factors, as we discussed above, the occurrence of CMI in Iraq/Afghanistan war veterans and in nondeployed veterans point to other basic causes. A common such factor is anthrax vaccination, which is compulsory in the U.S. armed forces. The epidemiological and clinical findings reported here, together with the laboratory findings reported previously [22,23,24], document the harmful effects of the anthrax vaccine and raise concerns about its long-term safety. A key contribution of the present study is the discovery of a subset of HLA Class II alleles which (a) are involved in the production of antibodies, (b) are present in vaccinated healthy veterans but (c) are absent in vaccinated veterans suffering from GWI. Given the estimated high binding affinity of the motif of these HLA molecules, this observation points to the lack of the ability of vaccinated veterans with GWI to produce antibodies against the anthrax vaccine antigen, a harmful factor [24]. This idea on the interplay of biological (toxicity of PA) and immunogenetic (HLA) factors for the development of GWI in vaccinated, immunogenetically vulnerable veterans is illustrated in the schematic diagram of Figure 5. Given that active military personnel are routinely anthrax vaccinated, and that 49.5% of veterans of the Iraq/Afghanistan wars suffer from CMI [43], the concerns above need to be urgently addressed.

## Figures and Tables

**Table 1:** Positive association of GWI with anthrax vaccine. *p*-values are 2-sided. See text for details.

		Anthrax Vaccination		
**GWI**		No	Yes	Total
	Absent	24	45	69
	Present	5	37	42
	Total	29	82	111
	% GWI	17.2%	45.1%	
	Statistics			
	Pearson χ^2^ = 7.080, *p* = 0.008			
	Likelihood ratio = 6.788, *p* = 0.006			
	Fisher’s exact test *p* = 0.008			
	φ = 0.353, *p* = 0.008			
	Common odds ratio = 3.947			
	Relative risk = 2.617			
	Absolute risk increase = 27.9%			

**Table 2:** The 18 HLA Class II alleles which occurred only in the anthrax-vaccinated veterans who did not develop GWI. Lowest percentile rank was the minimum estimated binding affinity of the allele to anthrax vaccine antigen PA across all 15-mer AA subsequences tested; values < 1 indicate high binding affinity. See text for details.

Index	HLA Allele	Lowest Percentile Rank
1	DPB1*06:01	0.030
2	DPB1*09:01	0.009
3	DPB1*10:01	0.050
4	DPB1*105:01	0.070
5	DPB1*126:01	0.050
6	DPB1*131:01	0.280
7	DPB1*14:01	0.160
8	DPB1*20:01	0.020
9	DPB1*23:01	0.040
10	DPB1*29:01	0.010
11	DQB1*05:02	0.160
12	DRB1*01:03	0.070
13	DRB1*04:04	0.030
14	DRB1*04:08	0.550
15	DRB1*08:03	0.760
16	DRB1*10:01	0.070
17	DRB1*11:04	0.580
18	DRB1*16:01	0.550

## Data Availability

The raw data supporting the conclusions of this article will be made available by the authors on request.

## Appendix A

**Table A1:** Amino Acid Sequence of Anthrax Protective Antigen. Labels are from https://www.uniprot.org/uniprotkb/P13423/entry (accessed on 20 August 2023).

P13423 · PAG_BACAN	Protective antigen (Bacillus anthracis)	764 AA

MKKRKVLIPLMALSTILVSSTGNLEVIQAEVKQENRLLNESESSSQGLLGYYFSDLNFQAPMVVTSSTTGDLSIPSSELENIPSENQYFQSAIWSGFIKVKKSDEYTFATSADNHVTMWVDDQEVINKASNSNKIRLEKGRLYQIKIQYQRENPTEKGLDFKLYWTDSQNKKEVISSDNLQLPELKQKSSNSRKKRSTSAGPTVPDRDNDGIPDSLEVEGYTVDVKNKRTFLSPWISNIHEKKGLTKYKSSPEKWSTASDPYSDFEKVTGRIDKNVSPEARHPLVAAYPIVHVDMENIILSKNEDQSTQNTDSQTRTISKNTSTSRTHTSEVHGNAEVHASFFDIGGSVSAGFSNSNSSTVAIDHSLSLAGERTWAETMGLNTADTARLNANIRYVNTGTAPIYNVLPTTSLVLGKNQTLATIKAKENQLSQILAPNNYYPSKNLAPIALNAQDDFSSTPITMNYNQFLELEKTKQLRLDTDQVYGNIATYNFENGRVRVDTGSNWSEVLPQIQETTARIIFNGKDLNLVERRIAAVNPSDPLETTKPDMTLKEALKIAFGFNEPNGNLQYQGKDITEFDFNFDQQTSQNIKNQLAELNATNIYTVLDKIKLNAKMNILIRDKRFHYDRNNIAVGADESVVKEAHREVINSSTEGLLLNIDKDIRKILSGYIVEIEDTEGLKEVINDRYDMLNISSLRQDGKTFIDFKKYNDKLPLYISNPNYKVNVYAVTKENTIINPSENGDTSTNGIKKILIFSKKGYEIG.
